# RNA polymerase supply and flux through the *lac* operon in *Escherichia coli*

**DOI:** 10.1098/rstb.2016.0080

**Published:** 2016-11-05

**Authors:** Bandar Sendy, David J. Lee, Stephen J. W. Busby, Jack A. Bryant

**Affiliations:** School of Biosciences and Institute of Microbiology and Infection, University of Birmingham, Birmingham B15 2TT, UK

**Keywords:** RNA polymerase, chromatin immunoprecipitation, promoter occupation, rifampicin, polymerase per second, polymerase density

## Abstract

Chromatin immunoprecipitation, followed by quantification of immunoprecipitated DNA, can be used to measure RNA polymerase binding to any DNA segment in *Escherichia coli*. By calibrating measurements against the signal from a single RNA polymerase bound at a single promoter, we can calculate both promoter occupancy levels and the flux of transcribing RNA polymerase through transcription units. Here, we have applied the methodology to the *E. coli* lactose operon promoter. We confirm that promoter occupancy is limited by recruitment and that the supply of RNA polymerase to the lactose operon promoter depends on its location in the *E. coli* chromosome. Measurements of RNA polymerase binding to DNA segments within the lactose operon show that flux of RNA polymerase through the operon is low, with, on average, over 18 s elapsing between the passage of transcribing polymerases. Similar low levels of flux were found when semi-synthetic promoters were used to drive transcript initiation, even when the promoter elements were changed to ensure full occupancy of the promoter by RNA polymerase.

This article is part of the themed issue ‘The new bacteriology’.

## 1. Introduction

Many bacteria rely on transcription regulation in order to adapt to fluctuating environments. This often involves the interaction of regulatory activator proteins at or near promoters, which results in recruitment of the DNA-dependent RNA polymerase (RNAP) and subsequent transcript initiation and gene expression. In contrast, when the regulatory proteins are repressors, access to the promoter is blocked and, hence, expression of the corresponding transcription unit is silenced [1–3]. Most experimental studies of bacterial gene regulation have relied on measurements of fold-induction or fold-repression of measured levels of transcripts or gene products. However, few studies have addressed directly the issue of the number of RNAP molecules that engage with individual transcription units, and, to date, most calculations of RNAP flux through genes are based on estimates that work backwards from measured levels of RNA synthesis [4–6]. Here, we describe a new approach to direct quantification of RNAP bound to the *Escherichia coli lac* operon and its promoter, exploiting chromatin immunoprecipitation (ChIP). Recall that ChIP, in combination with analysis of immunoprecipitated DNA, permits us to detect protein binding at any chromosomal locus [7], independent of function, and many investigators have used it to measure the distribution of RNAP across bacterial chromosomes [8–11]. Here, we exploit the properties of the drug rifampicin, which blocks RNAP bound at promoters [9,12–14], to calibrate our ChIP measurements. This allows an absolute measure of promoter occupancy and RNAP flux through downstream genes.

## 2. Results

### (a) Measurement of RNA polymerase flux though the *lac* operon

Formaldehyde treatment of cultures of *E. coli* efficiently cross-links RNAP to bound DNA targets [8,9]. Commercially available monoclonal mouse antibodies directed against the RNAP β subunit can then be used to immunoprecipitate RNAP from sonicated extracts of the cross-linked cells, and specific DNA targets can be quantified by PCR. We chose the well-characterized *E. coli* K-12 lactose (*lac*) operon to study RNA polymerase flux. Recall that the *lac* operon is expressed in a transcription unit from a promoter whose activity is repressed by the Lac repressor protein, and that induction requires a chemical inducer such as isopropyl β-d-1-thiogalactopyranoside (IPTG) [15,16]. To analyse RNAP flux through the *lac* operon, we used the lac0 pair of probes that samples the *lac* promoter, and the lac1–5 pairs of probes that sample approximately 300 base pair DNA sequences that are 518, 1421, 2308, 3691 and 4654 base pairs, respectively, downstream from the transcript start. Because each probe pair creates an amplicon that is a similar size, we can directly compare signal intensity between the different probes.

**Figure 1.**
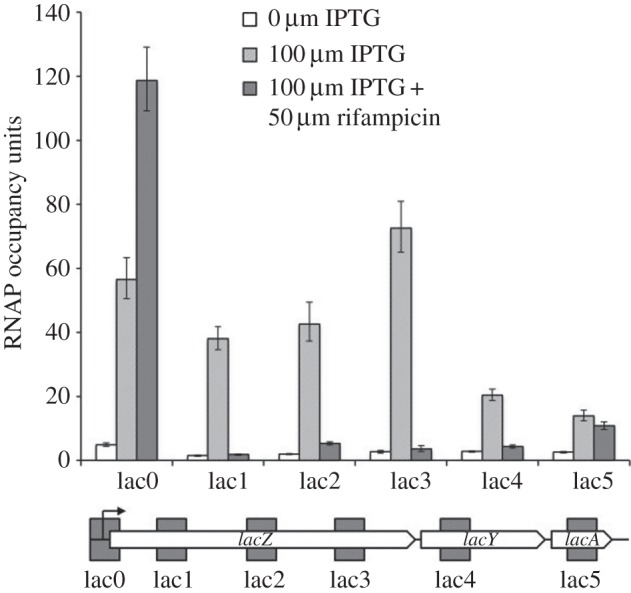
RNAP flux through the *lac* operon on the *E. coli* MG1655 chromosome. The figure shows experimentally measured RNAP occupancies at the *lac* promoter region (denoted lac0), or downstream regions (denoted lac1–5), illustrated in the sketch of the operon (approximately to scale). The probes are located from position −147 to +123 (lac0), position +518 to +781 (lac1), position +1421 to +1686 (lac2), position +2308 to +2575 (lac3), position +3691 to 3949 (lac4) and position +4654 to 4916 (lac5), all positions being with respect to the *lac* operon transcript start site. Cell cultures were grown and treated with formaldehyde, as described in the Material and methods section. Total DNA with cross-linked proteins was extracted and sonicated, and fragments cross-linked to RNAP were purified by immunoprecipitation. RNAP occupancy was measured by a ChIP–qPCR protocol. The figure illustrates measurements from cells grown with or without the inducer IPTG and with or without rifampicin, as indicated by the different shadings.

*Escherichia coli* K-12 strain MG1655, growing exponentially in medium, either with or without IPTG, was subject to our ChIP protocol (see Material and methods section for details, and electronic supplementary material, table S1 for probe sequences), and figure 1 shows quantification of the immunoprecipitated DNA at different loci in the *lac* operon (probed with the lac0–5 probes). The data show that the inclusion of IPTG in the bacterial growth media triggers a more than a 100-fold increase in levels of imunoprecipitated DNA, confirming that RNAP association with the *lac* operon is regulated by the Lac repressor. Accepting that the level of immunoprecipitated DNA corresponding to each probe reflects the amount of transcribing RNAP associated with the chromosomal DNA corresponding to each probe, the data argue that, at least for the first 2000 base pairs of the operon, RNAP levels remain constant, while they decline towards the end of the operon, presumably contributing to polarity effects [17]. Some quantitative differences seen with certain fragments, for example, with the lac3 probes, are likely owing to pause sequences [18,19].

In order to calculate the absolute numbers of RNA polymerase molecules associated with the *lac* operon from the data in figure 1, we needed to measure the quantity of immunoprecipitated DNA that results from the binding of a single RNAP molecule. To do this, we exploited the property of rifampicin to block initiating RNAP at promoters and to inhibit transcript elongation [9]. Hence, rifampicin was added to MG1655 cells growing in the presence of IPTG, and figure 1 shows quantification of immunoprecipitated DNA at the different *lac* operon loci, probed with the lac0–5 probes. As expected, rifampicin causes a sharp decrease in the levels of immunoprecipitated DNA corresponding to the lac1–5 probes, but an increase with the lac0 probes. If we take the measured signal with the lac0 probe as indicative of a single promoter-bound RNAP, then we can deduce that, during induction in our growth conditions, the *lac* promoter is approximately 50% occupied, which is consistent with experimental data showing that the activity of the *E. coli lac* promoter is limited by the recruitment of RNAP [20,21]. Furthermore, the data permit an estimate of the flux of RNAP through the *lac* operon. Messenger elongation by RNAP in bacteria is known to proceed, on average, at 20–50 bases per second [22–24]. Because the DNA segment corresponding to each of the lac1–5 probes consists of approximately 300 base pairs, which would take at least 6 s to transcribe, approximately 33% observed occupancy by RNAP implies that a transcribing RNAP must arrive on average no more frequently than once every 18 s (3 × 6). This unexpected low level is likely owing to the time that individual RNAP molecules can take to escape from the promoter [25–27].

### (b) RNA polymerase supply at the *lac* promoter is location-dependent

**Figure 2.**
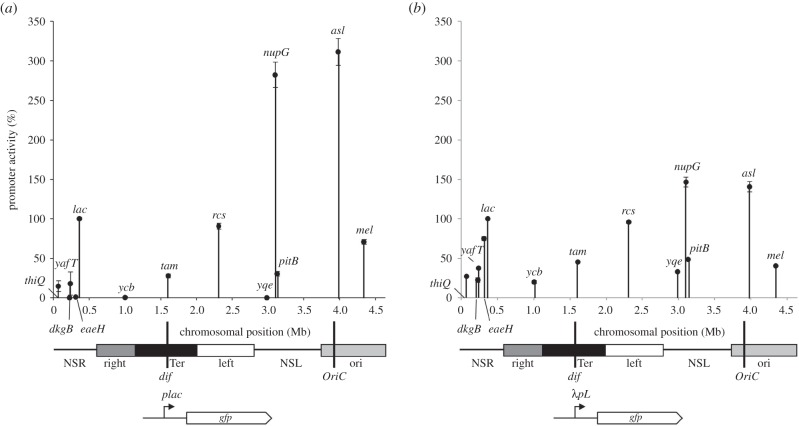
Chromosome position effects on activities of the *lac* (*a*) and *λpL* (*b*) promoters. The figure shows experimentally determined measurements of the expression of *lac* and *λpL* promoter::*gfp* fusions, at different locations on the *E. coli* chromosome. Fluorescent output from the reporter cassette was measured during growth in the presence of 100 µM IPTG, and is represented on the *y*-axis. Chromosomal positions of the reporter cassette are represented on the *x*-axis and denoted in the figure by the name of a neighbouring gene. Below each chart is a linear schematic of the *E. coli* genome, with the origin of replication (*OriC*), terminus (*dif*), macrodomains and non-structured regions (NSR, right, Ter, left, NSL, ori) shown as previously reported [30].

In a previous study, we found that the measured activity of the *lac* promoter in *E. coli* strain MG1655 was dependent on its chromosomal location [28]. To show this, we constructed a portable *lac* promoter::green fluorescent protein (*gfp*) cassette that we inserted at different chromosomal locations. We found that expression varied by up to 200-fold according to location, and, using ChIP, we showed that the measured differences were due to different levels of RNAP associated with the *gfp* gene. Because *lac* promoter activity is limited by RNAP recruitment [20], we reasoned that the differences could be caused by the concentration of available RNAP differing from one chromosomal location to another. Hence, to test this, we replaced the *lac* promoter with the bacteriophage *λ* major leftward promoter (*λP_L_*), whose activity is known to be limited by RNAP escape rather than recruitment [29]. Figure 2 shows the results of an experiment where we compared *gfp* expression from either our *lac* promoter::*gfp* or *λP_L_*::*gfp* fusions inserted into the MG1655 chromosome at different specific locations. The data show that observed differences in expression are much smaller with the *λP_L_*::*gfp* fusions than with the *lac* promoter::*gfp* fusions, and this is consistent with the suggestion that the effective concentration of RNAP differs according to location along the *E. coli* chromosome.

### (c) Promoter determinants alter RNA polymerase recruitment and RNA polymerase escape

**Figure 3.**
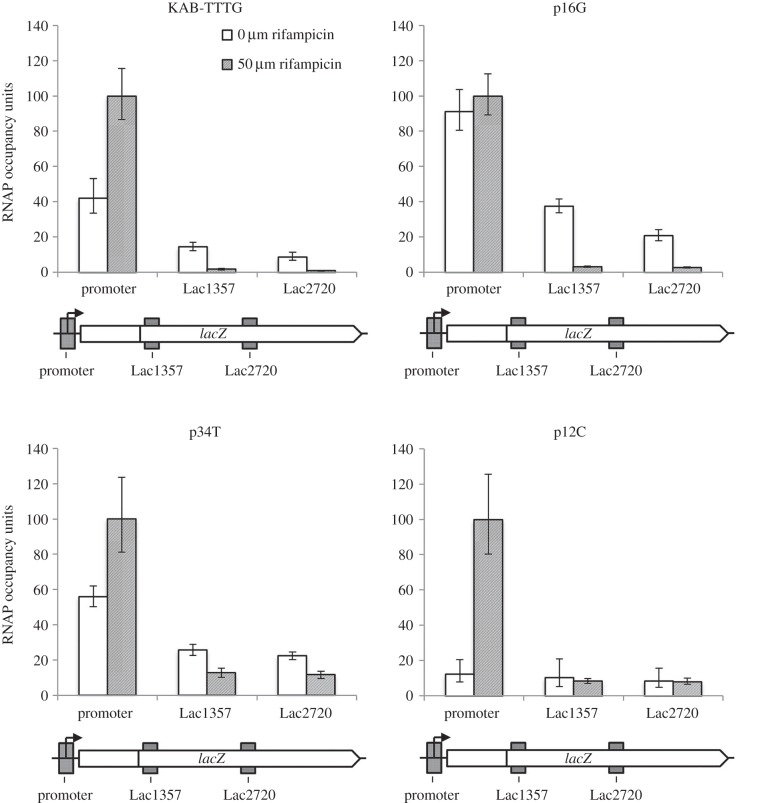
RNAP flux through the *lac* operon controlled by synthetic promoters. The figure shows experimentally determined levels of RNAP occupancy at the promoter and downstream regions Lac1357 and Lac2720, measured by ChIP–qPCR, during exponential growth in the presence of absence of rifampicin, as indicated by the different shading. The positions of probe regions are shown on the schematic diagrams (approximately to scale). RNAP occupancy of each promoter in the presence of rifampicin is taken as 100% occupied and other figures normalized accordingly. Data are shown for the KAB-TTTG promoter and three mutant derivatives: p16G (an extended −10 element ‘up’ mutant), p34T (a −35 element ‘up’ mutant) and p12C (a −10 element ‘down’ mutant).

Although the *E. coli lac* operon promoter is often adopted as a paradigm, we wanted to compare results with an unrelated promoter, and so we used a previously constructed set of promoter::*lac* fusions [31], where the promoters carry different combinations of −35, extended −10 and −10 elements upstream of the *galP1* transcript start region. In an initial experiment, we selected the KAB-TTTG promoter that carries the −35 element TAGACA (consensus is TTGACA), an extended −10 element of TTTG (consensus is TGTG) and a −10 hexamer of TATGGT (consensus is TATAAT). Figure 3 illustrates ChIP data from an experiment run either with or without rifampicin, from which, as before, RNAP occupancy can be calculated. Surprisingly, the data reveal occupancy and flux levels that are similar to the induced *lac* promoter. Hence, promoter occupation by RNAP, as judged by the ratio of signal without rifampicin to with rifampicin, is approximately 40%, whereas occupancy of the downstream DNA segments corresponding to the Lac1357 and Lac2720 probes, ranges from 10% to 20%, which would correspond to an RNAP flux of one every 30 s.

Because it is well established that promoter −35 and extended −10 elements contribute to the recruitment of RNAP at bacterial promoters [32,33], we repeated the experiment with derivatives of the KAB-TTTG promoter carrying the p34T point mutation that creates a consensus −35 element (TTGACA), or the p16G mutation that creates a consensus extended −10 element (TGTG). As a control, we also used a derivative of the KAB-TTTG promoter with the p12C mutation that creates a corrupted −10 element (CATGGT). The results, illustrated in figure 3, show that recruitment of RNAP is reduced by the −10 element mutation. In contrast, recruitment of RNAP to the promoter is increased to approximately 50% by the consensus −35 element, and to nearly 100% by the consensus extended −10 element. However, for both promoters, the increase in occupancy leads to only modest increases in RNAP flux through the downstream-transcribed DNA.

## 3. Discussion

We have developed a simple method, based on ChIP with *E. coli*, for quantifying the binding of RNAP *in vivo* to any specific segment of DNA. For promoter regions, we can directly measure occupancy by RNAP, whereas for regions within transcription units, we can deduce the flux of RNAP. The method exploits rifampicin that specifically targets RNAP and blocks it in open complexes at promoters. Here, we make the assumption that formaldehyde equally efficiently cross-links rifampicin-blocked RNAP, RNAP that is bound and paused at promoters, and elongating RNAP, to cognate DNA targets. This appears reasonable as RNAP has a large molecular mass, and makes intimate contacts with the DNA template in all three situations.

Our results are consistent with previous observations that transcript initiation at the *lac* operon promoter is limited by RNAP recruitment [20,21]. We believe that this explains, at least in part, our previous observation that the expression of a *lac* promoter::*gfp* fusion differs according to its location on the *E. coli* chromosome [28], because the activity of a promoter that is limited by RNAP recruitment will depend on the local concentration of available RNAP. Hence, we suggest that, according to its position on the *E. coli* chromosome, the promoter will sample different locations, including locations where the effective concentration of RNAP is higher or lower. Consistent with this, a recent live-cell super-resolution microscopy study of RNAP in *E. coli* showed that the vast majority of non-transcribing RNAP molecules that were ‘searching’ for promoters were DNA-bound [34]. Interestingly, we found that Lac repressor-mediated repression of the *lac* operon promoter is not dependent on location [28]. From this, we deduce that diffusion of the Lac repressor ensures that its effective concentration is the same at all locations within the *E. coli* cell, whereas diffusion of larger RNAP molecules is constrained, and this is consistent with calculations of macromolecular mobility in bacteria [35].

The low measured flux of RNAP through the *lac* operon is consistent with previous observations, from both *in vitro* [26,27] and *in vivo* [36] studies, that the transition of RNAP from the transcriptionally competent open complexes to the elongating complex, via the promoter escape phase, is not simple and can be slow and rate-limiting. We observe low flux of one RNAP every 18–50 s, irrespective of whether transcription was being driven by the *lac* promoter or by genetically engineered promoters, even when the promoter is fully occupied. This underscores that promoters, as well as being drivers of transcription, are also bottlenecks, and delays to RNAP result in reduced flux through downstream-transcribed sequences [36,37]. Contributing reasons for delays include pauses owing to scrunching [38–40], pauses owing to disengagement of various RNAP determinants with promoter elements [32,33,41] and sigma factor-mediated pauses early in the elongation phase [42,43]. Additionally, it may well be that there are topological and mechanical reasons why transcribing RNAP molecules must be well separated, but, to date, these are speculative and poorly understood.

We are aware that the measured rates of RNAP flow that we report here are surprisingly low, and depend critically on estimates of RNA chain growth rates. Hence, it is worth underscoring that measured *in vivo* rates of RNA chain elongation [22–24] are corroborated by single molecule studies of RNA chain growth *in vitro* [44], and that previous estimates of RNAP flux, defined by synthetic biologists in terms of polymerase per second (PoPS) units, are consistent with our findings [5,45]. The prime motive for initiating this project was the need, perceived by the synthetic biologists, to provide robust characterization for ‘parts’ that could be used in novel circuits. Taken together, our results argue that full and robust characterization might not be possible, and, for many promoters, their ‘performance’ is context-dependent. Recent insights into transcript initiation and elongation, confirmed here by the low measured levels of RNAP flux through the *lac* operon, contradict the simple view that promoters are simply devices that ‘feed’ RNAP into transcription units [37]. Hence, while ‘parts’ such as the *lac* promoter have many uses in synthetic biology, their full exploitation will require considerable extension of our current knowledge base.

## 4. Material and methods

### (a) Bacterial strains, plasmids and growth conditions

The experiments analysing the flux of RNAP through the chromosomal *lac* operon were completed using *E. coli* K-12 strain MG1655 [46], whereas the synthetic promoter experiments were conducted using a Δ*crp* derivative of strain M182 [47], with the promoter::*lac* fusion carried on the low copy number broad host range *lac* expression vector, pRW50 [31]. Fragments carrying the different KAB promoter derivatives were previously described [31,48]. The promoter derivatives are denoted pNX, where N is the position of the substitution upstream from the transcript start, and X is the substituted base on the non-template strand.

For the ChIP assays, triplicate single colonies were used to inoculate Luria Bertani (LB) media, supplemented with 35 µg ml^−1^ tetracycline, where appropriate, and incubated for 16 h at 37°C with aeration. Cells were then subcultured into fresh media to a final OD_650_ of 0.03, then incubated at 37°C with aeration until an OD_650_ of 0.4 was reached. The growth conditions used for fluorescence assays were the same, except M9 minimal media supplemented with 2 mM MgSO_4_, 0.1 mM CaCl_2_, 0.1% casamino acids, 0.3% fructose and 100 µM IPTG was used [28].

### (b) Construction of plasmids, chromosomal recombination and green fluorescent protein measurements

To construct the gene doctoring donor plasmids required to insert the *λP_L_*::*gfp* fusion into the genome of *E. coli* MG1655, an oligodeoxynucleotide was synthesized to encode an *Eco*RI restriction target site and the *λP_L_* G-12 T up-mutant *λ* leftward promoter fused to the ribosome binding site of the *lac*Z gene. This ribosome binding site was used in order to make the fusion comparable to previously used *lac* promoter::*gfp* fusion [28]. This oligonucleotide primer was then used with a primer downstream of the *Hin*dIII site in the pJB plasmids to create an *Eco*RI–*Hin*dIII *λP_L_* promoter fragment, which was subsequently cloned into *Eco*RI–*Hin*dIII digested pJB plasmids containing the appropriate homology regions for insertion into the chromosomal targets used previously [28]. Insertion of the *λP_L_* promoter::*gfp* fusion into the target chromosomal loci was achieved exactly as described previously by the gene doctoring chromosome recombineering method [28,49], and fluorescence assays were run using the growth conditions described above.

### (c) Chromatin immunoprecipitation and quantitative real-time PCR analysis

Chromatin immunoprecipitation (ChIP) and quantitative real-time PCR (qPCR) were performed as before, using commercial mouse monoclonal antibody for the RNAP β subunit (Neoclone no. W0002) to immunoprecipitate DNA cross-linked to RNAP [8,28,50]. Overnight cultures were used to subculture into fresh LB to a final OD_650_ of 0.025, and incubated at 37°C, with aeration, until an OD_650_ of 0.4 was reached. When appropriate, rifampicin was added to the culture to a final concentration of 50 µM, and incubated for 15 min, which is sufficient to trap RNAP molecules at promoters [8,9]. Cells were cross-linked by addition of formaldehyde to a final concentration of 1%, and incubated for a further 20 min at 37°C. After the ChIP protocol, immunoprecipitated DNA was quantified by qPCR, using the Agilent Technologies Stratagene Mx3005P machine and the Agilent Brilliant III Ultra-fast SYBR Green qPCR master mix, and this permitted calculation of RNAP occupancy units for each amplicon. Oligonucleotide primers were designed to amplify approximately 300 bp regions with the same PCR efficiency, either at promoter regions or within the *lac* operon (primer sequences are listed in electronic supplementary material, table S1). Control primers were used to amplify transcriptionally silent control regions. Enrichment of ChIP samples for RNAP binding was calculated relative to the transcriptionally silent control regions, as previously described [50], with the samples from rifampicin-treated cells used to define 100% occupation.

## Supplementary Material

Supplementary Table 1
