# Supplementary material for: RNA polymerase supply and flux through the lac operon in Escherichia coli
Source: Philos Trans R Soc Lond B Biol Sci. 2016 Nov 5;371(1707):20160080. doi: 10.1098/rstb.2016.0080 (PMC5052750; doi:10.1098/rstb.2016.0080)
Supplement: Supplementary Table 1 [file rstb20160080supp1.pdf]

Supplementary Table 1: Primer sequences for qPCR

Probe	Oligo name	Oligodeoxyribonucleotide sequence (5'-3')
lac0	Lac0 Fwd	GCGTTGGCCGATTCATTAATGCAGC
	Lac0 Rev	CAAGGCGATTAAGTTGGGTAACGCCAG
lac1	Lac1 Fwd	GGTTACGGCCAGGACAGTCG
	Lac1 Rev	CACATCTGAACTTCAGCCTCCAGTAC
lac2	Lac2 Fwd	GAATCAGGCCACGGCGCTA
	Lac2 Rev	AACCGCCAAGACTGTTACCCATC
lac3	Lac3 Fwd	GCAATTTAACCGCCAGTCAGGC
	Lac3 Rev	TGATGCTGCCACGCGTGAG
lac4	Lac4 Fwd	TGCACTCATCCTCGCCGTTTTACTC
	Lac4 Rev	CCAAATACCCGCGTACCCTGTTC
lac5	Lac5 Fwd	GTAGGGGAAAACGCCTGGGTAG
	Lac5 Rev	CCACATGACTTCCGATCCAGACG
BglB	bglB Fwd	GGATCAAGCCGCTGGTAACG
	bglB Rev	CTGGCAACCAGTTGATGGTGGA
Promoter	Promoter Fwd	TCGCAAGGACGAGAATTTCC
	Promoter Rev	GTTTTTCAGCAGGTCGTTG
Lac1357	lacZ1357 Fwd	GACAGTATCGGCCTCAGGAA
	lacZ1357 Rev	AACGTCGTGACTGGGAAAAC
Lac2720	lacZ2720 Fwd	TCTCTCCAGGTAGCGAAAGC
	lacZ2720 Rev	TAATCACGACGCGCTGTATC
Control	Control Fwd	CAGTCCATCAGGTAATTGCCG
	Control Rev	GCGCAAACTGTTAATGCTGG

The Table lists the base sequences of oligo pairs used to quantify specific DNA segments. The lac0 probe targets the *E. coli lac* operon promoter region, whilst the lac1-5 probes target different segments of the *lac* operon (illustrated in Figure 1). The BglB probe serves as a control, targeting a non-expressed segment of the *E. coli* chromosome. The probes denoted 'Promoter' amplify promoter fragments cloned in the pRW50 low copy number broad host range *lac* expression vector [30], and the Lac1357 and Lac2720 probes target *lac* operon sequences downstream of these cloned promoters (illustrated in Figure 3). The probe denoted 'Control' target a non-expressed segment of pRW50.
